# Integrins as Therapeutic Targets for SARS-CoV-2

**DOI:** 10.3389/fcimb.2022.892323

**Published:** 2022-04-29

**Authors:** Timothy E. Gressett, Danielle Nader, Juan Pablo Robles, Tione Buranda, Steven W. Kerrigan, Gregory Bix

**Affiliations:** ^1^ Tulane University School of Medicine, Clinical Neuroscience Research Center (CNRC), New Orleans, LA, United States; ^2^ Department of Neurology, Tulane University School of Medicine, New Orleans, LA, United States; ^3^ Tulane Brain Institute, Tulane University, New Orleans, LA, United States; ^4^ RCSI University of Medicine and Health Sciences, School of Pharmacy and Biomolecular Sciences (PBS), Dublin, Ireland; ^5^ Instituto de Neurobiología, Universidad Nacional Autónoma de México (UNAM), Juriquilla, Mexico; ^6^ University of New Mexico Health Sciences Center (HSC), Department of Pathology, Albuquerque, NM, United States

**Keywords:** integrins, SARS-CoV-2, therapeutic, RGD, ATN-161, cilengitide

## Introduction

Severe acute respiratory syndrome coronavirus 2 (SARS-CoV-2) is an enveloped, positive-sense, single-stranded RNA virus of the genus Betacoronavirus. Its genome is composed of four structural proteins known as spike (S), envelope (E), membrane (M), and nucleocapsid (N), of which E, M, and N are integrated into the viral envelope. The S glycoprotein, which protrudes from the surface of mature virions as a spike, is essential for virus attachment, fusion, and entry into the host cell.

**Figure 1:**
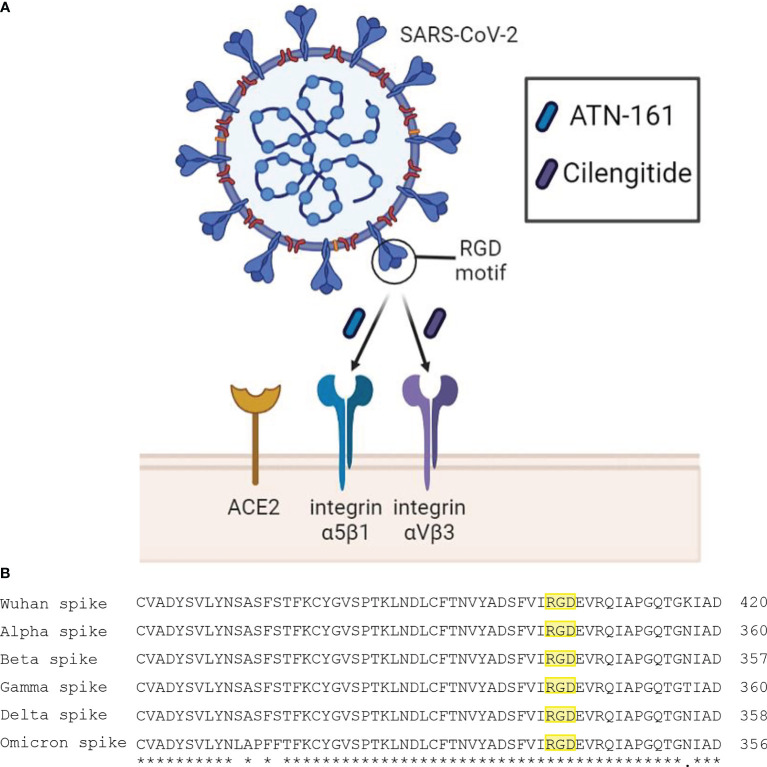
**(A)** Schematic diagram of SARS-CoV-2 interaction with proposed receptors and therapeutics. Integrins α5β1 and αVβ3 have been reported to bind the RGD motif of the spike protein. Peptide compounds ATN-161 and Cilengitide target these integrins and have displayed efficacy in reducing SARS-CoV-2 infection and spike-mediated endothelial dysfunction. **(B)** Multiple sequence alignment using EMBL-EBI Clustal Omega tool between spike proteins of the SARS-CoV-2 Wuhan wild type, and variants of concern Alpha, Beta, Gamma, Delta, and Omicron. The symbols (*) and (.) indicate conserved and weakly similar amino acids, respectively. The RGD motif is highlighted in yellow, where it is preserved across all major variants.

While the relationship between the spike protein of SARS-CoV-2 and the angiotensin-converting enzyme 2 (ACE2) receptor has been readily established, the S1 subunit also contains a solvent-exposed arginine-glycine-aspartic acid (RGD) binding motif that is predominantly recognized by integrins, specifically α_5_β_1_ and α_V_β_3_ (Sigrist et al., 2020; Tresoldi et al., 2020). These integrins, which are primarily expressed on vascular endothelial cells, are part of a large family of heterodimeric transmembrane receptors containing an α and a β subunit and are devoted to cell adhesion to the extracellular matrix and other signaling effects and functions to include the immune response (Hynes, 2002). Blockade of SARS-CoV-2 binding to α_5_β_1_ and α_V_β_3_ integrins using the small peptides ATN-161 and Cilengitide, respectfully, has been shown to reduce viral infectivity *in vivo* and attenuate vascular inflammation (Amruta et al., 2021; Nader et al., 2021; Robles et al., 2022). We therefore propose an urgent examination into the therapeutic potential of integrins as therapeutics targets for SARS-CoV-2 (**Figure 1**).

## Mechanism of Spike Protein-Mediated Viral Entry

The S glycoprotein—or “spike protein”—consists of two non-covalently associated subunits. The S1 subunit binds to receptors, while the S2 subunit anchors the spike protein to the virion membrane to mediate membrane fusion. SARS-CoV-2 fusion occurs mainly after the ACE2 receptor engages the S1 subunit of the spike protein, exposing a protease cleavage site on the S2 subunit (S2’). Cleavage at S2’ by the transmembrane protease serine 2 (TMPRSS2) at the surface, or by cathepsins in endosomal compartments, triggers the fusion of the virus (Jackson et al., 2021).

Although canonically known to bind to ACE2, emerging evidence has shown that the S glycoprotein binds to integrins which influences SARS-CoV-2 infectivity (Makowski et al., 2021; Simons et al., 2021; Staufer et al., 2022). In addition, viruses such as SARS-CoV-2 have evolved to use integrins to facilitate cell entry, extending tissue tropism and infectivity (Hussein et al., 2015). Integrins, therefore, are potentially attractive targets for blocking viral infection.

## Integrins Are Receptors for SARS-COV-2

SARS-CoV-2 is unique among all other Betacoronaviruses in that it has a novel K403R substitution in the distal tip of the spike protein compared to SARS-CoV-1 (Sigrist et al., 2020). This sequence encodes an RGD motif which is recognized by RGD-binding integrins. Evidence of direct binding of spike protein to integrins has been reported and is successfully reduced upon adding RGD-blocking compounds or integrin-targeting monoclonal antibodies in several cell lines which express α_5_β_1_ and α_V_β_3_, such as human umbilical vascular endothelial cells, colonic cells, and primary derived aortic endothelial cells (Nader et al., 2021; Robles et al., 2022), important as RGD-motif integrins are differentially expressed on endothelial cells developmentally and assume this same phenotype during cytokine mediated-injury (Gonzalez and Medici, 2014; Wang et al., 2018). Interestingly, besides the RGD motif, additional potential integrin-binding motifs may have equal or better accessibility for integrin binding on not only α_5_β_1_ and α_V_β_3_ but on several other integrin subunits (Beaudoin et al., 2021), suggesting that potential non-RGD interactions on integrins may also contribute to SARS-CoV-2 infectivity. Perhaps even more interesting is that emerging SARS-CoV-2 mutations of concern have conspicuously left the RGD motif untouched (Makowski et al., 2021), which supports the hypothesis that integrins may contribute to SARS-CoV-2 viral entry and infectivity beyond ACE2 binding alone.

Emerging data also suggests that the mutations within the Omicron variant facilitate a receptor-binding domain that allows more accessibility to the RGD motif, which may ultimately enhance integrin binding (Hossen et al., 2022) and be associated with higher transmission rates than other variants of concern. Mapping the tissue distribution of integrins also highlights its potential as an alternative receptor. While ACE2 has low expression in alveolar, bronchial, and pulmonary endothelial cells, high levels of integrins have been noted in both upper and lower epithelial and endothelial respiratory cells (Meng et al., 2022), a primary cellular target for SARS-CoV-2. In addition, integrins appear to be upregulated in COVID-19 affected patients (Wu et al., 2020; Calver et al., 2021). Taken together, integrins may be significant regulators of SARS-CoV-2 infection and potential determinants for tissue tropism and COVID-19 severity.

## Productive Infection Requires Integrins

The putative integrin-dependent infection mechanism of SARS-CoV-2 is thus far unknown. However, ebolavirus (Schornberg et al., 2009) and reoviruses (Maginnis et al., 2008) rely on an integrin α_5_β_1_-dependent and clathrin-mediated endocytosis that delivers their viral cargo to endo-lysosomes, where the S2’ is cleaved by cathepsins to facilitate fusion between virus and host membranes (Maginnis et al., 2008; Schornberg et al., 2009). This process is mediated by the NPxY motif in the cytoplasmic tail of the β subunit of integrins, which recruits talin, an actin-binding focal adhesion protein essential for integrin activation, and clathrin-adaptor proteins required for delivery to endosomes and lysosomes (Caswell et al., 2009). Studies on reovirus infection in spinner-adapted fibroblast cells have demonstrated that mutations in the NPxY motif on β_1_ integrins result in dysfunctional trafficking and non-productive infection (Maginnis et al., 2008). Likewise, steric inhibition of talin-binding to the NPxY motif on the ß cytoplasmic tail with the cell membrane permeable small-peptide mP13 blocks productive infection of Sars-CoV-2 (Simons et al., 2021). This highlights the critical role of integrins in SARS-CoV-2 productive infection.

## Integrins Are Involved in Vascular Dysregulation

In endothelial cells, integrins regulate barrier integrity by mediating intracellular signaling cascades triggered upon ligand binding. Through its RGD motif, SARS-CoV-2 spike protein induces significant cellular permeability and vascular dysregulation through the downregulation or internalization of junction proteins, to include VE-Cadherin endothelial adherens junction protein, JAM-A tight junctional protein, Connexin-43 gap junctional protein, and Platelet endothelial cell adhesion molecule-1 (PECAM-1) in primary mouse brain microvascular endothelial cells (Biering et al., 2021; Raghavan et al., 2021). Strikingly, integrin α_5_β_1_ activation by spike protein induces an endothelial inflammatory phenotype characterized by increased leukocyte attachment to the endothelium and the expression of inflammatory cytokines and coagulation factors (Robles et al., 2022). Blocking integrin receptors using antagonists which target α_V_β_3_ or α_5_β_1_ integrins successfully rescue barrier function as measured by VE-Cadherin and FITC-Dextran while additionally reducing immune leukocyte adhesion and permeability (Nader et al., 2021; Robles et al., 2022). The vasculopathy experienced in COVID-19 affected individuals, therefore, may very well be likely attributed to SARS-CoV-2 spike protein exploitation of integrins.

## Therapeutics for Targeting Integrins

Several integrin-targeting formulations have been safely administered to humans to treat various diseases, which could be repurposed to treat SARS-CoV-2 infection. These include the antibody natalizumab, which targets α_4_β_1_/β_7_ integrins for the treatment of Crohn’s disease and multiple sclerosis (MS), the small molecule tirofiban, which targets α_IIb_β_3_ integrins and is an anti-platelet therapy for the treatment of acute coronary syndrome, the experimental cancer therapy cyclic peptide Cilengitide which targets α_v_ integrins, and the experimental cancer and stroke therapy small peptide ATN-161, which primarily targets α_5_β_1_ but also can inhibit α_V_β_3_ integrin.

Natalizumab has shown favorable outcomes for COVID-19 patients while treating their multiple sclerosis, supporting the hypothesis that targeting integrins with prophylactic natalizumab might reduce SARS-CoV-2 host cell infection and subsequent replication in humans (Aguirre et al., 2020). Likewise, Tirofiban has improved hypoxemia in severe COVID-19 patients with hypercoagulability (Viecca et al., 2020). The cyclic RGD-based compound Cilengitide has demonstrated a high affinity for integrin α_V_β_3_ (Kapp et al., 2017), and in cultured human endothelial cells, has also been shown to inhibit SARS-CoV-2 spike protein binding (Nader et al., 2021). Finally, our group has validated ATN-161 as an inhibitor of spike protein-mediated cell infection *in vitro* (Beddingfield et al., 2021). Then, for the first time, demonstrated the *in vivo* therapeutic potential of an integrin-based inhibition therapy for SARS-CoV-2 infection. Our results showed that ATN-161 administered post-SARS-CoV-2 infection limited viral load in the lungs, improved lung histology, and reduced the inflammatory potential in SARS-CoV-2 susceptible k18-hACE2 transgenic mice (Amruta et al., 2021).

Since integrins are critical receptors for multiple cellular functions, potential side effects of integrin-targeting drugs, especially for antibody-based drugs, warrant further discussion. Natalizumab carries the risk of inducing progressive multifocal leukoencephalopathy (Babaesfahani et al., 2022), while Tirofiban carries a risk of hematuria and hemorrhage (Iqbal et al., 2022). Finally, antibody-based drugs may also have a cost concern, which, relative to a SARS-CoV-2 antibody-based therapy, might not be as cost effective or efficient.

ATN-161 is a five–amino-acid peptide derived from the synergy binding region of fibronectin to α_5_β_1_ and has shown excellent safety profiles in intravenous infusion at multiple dose escalations, cycles, and timepoints during Phase I clinical trials in solid tumor glioblastoma with no significant toxicity reported at the maximum administered dose (Cianfrocca et al., 2006). Likewise, Cilengitide, which selectively blocks activation of α_v_β_3_, shows a similar safety profile in patients with glioblastoma (Scaringi et al., 2012). Targeted integrin therapies towards SARS-CoV-2, specifically those involving α_5_β_1_ and α_v_β_3,_ should therefore be seriously considered.

## An Integrin-Based Treatment for SARS-COV-2

Although comparative analysis of how integrins may participate in SARS-CoV-2 pathophysiology has yet to be conducted, we believe that integrin α_5_β_1_ and α_V_β_3_ may be promising targets to mediate productive infection in SARS-CoV-2. Besides robust experimental evidence, other considerations support this hypothesis. The cytoplasmic tail on α_5_β_1_ has *in-cis* interactions between ACE2, which provide synergistic upregulation to cell adhesion signaling (Clarke et al., 2012), which may ultimately enhance SARS-CoV-2 infectivity and thus explain the reduction of pathophysiological outcomes after inhibiting this receptor *in vivo* (Amruta et al., 2021). In addition, several studies have shown that ACE2 and integrin β_1_ are upregulated in tandem due to inflammatory cytokines released in severe COVID-19 and in several health comorbidities that reduce clinical outcomes, to include hypertension and hyperlipidemia, diabetes, chronic pulmonary diseases, old age, and smoking (Jackson et al., 2021).

Both ATN-161 and Cilengitide may be promising therapeutics as integrin inhibitors in treating SARS-CoV-2 infection. In addition to their excellent clinical safety profile, they are safe to administer systemically and may even be delivered as an intranasal spray for more targeted delivery to the respiratory tract as either a pre- or post-exposure prophylactic. Furthermore, a potential advantage for using ATN-161 for treating SARS-CoV-2 infection is its proclivity for binding to and inhibiting activated—that is, bound with viral spike protein or primed for binding—versus inactive forms of α_5_β_1_ integrin (Beddingfield et al., 2021), thereby limiting its potential for off-target effects. Indeed, in addition to post-exposure prophylaxis with antivirals or monoclonal antibodies to prevent SARS-CoV-2 infection from progressing to severe COVID-19 in certain at-risk individuals, integrin inhibitors, such as ATN-161 or Cilengitide, may represent a novel pre-exposure prophylactic approach to preventing SARS-CoV-2 and are worthy of further study.

## Discussion

While vaccination combined with other mitigation strategies such as mask-wearing, avoiding large indoor crowds in poorly ventilated locations, and social distancing continue to be the most effective COVID-19 preventative approaches, new therapeutic strategies remain attractive. The evidence clarifies that the spike protein of SARS-CoV-2, through its integrin-binding RGD motif, allows integrins to mediate SARS-CoV-2 infection and spike-mediated endothelial dysfunction (Biering et al., 2021; Robles et al., 2022). What remains is to delineate how integrins participate in cell entry and trafficking of the virus, and ultimately, determine whether specific integrins, such as α_5_β_1_, αVβ3, or a group of integrins, play a crucial role in mediating infection. Clarifying these mechanisms may determine whether a specific integrin inhibitor, or perhaps an integrin inhibitor “cocktail” might be readily effective and repurposed to prevent and treat SARS-CoV-2 infection and related COVID-19 clinical sequelae.

## Author Contributions

All authors listed have made a substantial, direct, and intellectual contribution to the work and approved it for publication.

## Conflict of Interest

The authors declare that the research was conducted in the absence of any commercial or financial relationships that could be construed as a potential conflict of interest.

## Publisher’s Note

All claims expressed in this article are solely those of the authors and do not necessarily represent those of their affiliated organizations, or those of the publisher, the editors and the reviewers. Any product that may be evaluated in this article, or claim that may be made by its manufacturer, is not guaranteed or endorsed by the publisher.
